# hsa_circ_0008234 inhibits the progression of lung adenocarcinoma by sponging miR-574-5p

**DOI:** 10.1038/s41420-021-00512-1

**Published:** 2021-05-28

**Authors:** Wei Jiang, Yaozhou He, Zijian Ma, Yu Zhang, Chengpeng Zhang, Nianpeng Zheng, Xing Tang

**Affiliations:** 1 Department of Thoracic Surgery, The First Affiliated Hospital of Soochow University, Suzhou, Jiangsu Province P. R. China; 2 Department of Thoracic Surgery, The First Affiliated Hospital of Nanjing Medical University, Nanjing, Jiangsu Province P. R. China

**Keywords:** Non-coding RNAs, Non-small-cell lung cancer, Cell growth, Apoptosis

## Abstract

circRNAs are a novel type of noncoding RNA (ncRNA) that have been identified as an important regulator of gene expression and play a part in the progression of various diseases. However, the function of circ_0008234 in lung adenocarcinoma (LUAC) remains unknown. Through the GEO (Gene Expression Omnibus) database, circ_0008234 was first found to be downregulated in LUAC tissues. It could inhibit cell growth and accelerate apoptosis in vitro and in vivo. In terms of its possible mechanism, circ_0008234 mainly was present in the cytoplasm and competed with miR-574-5p to regulate RND3 (Rho family GTPase 3). Our results revealed that circ_0008234 inhibited the progression of LUAC through a competing endogenous RNA (ceRNA)-based mechanism and provided potential biomarkers and therapeutic targets for LUAC treatment.

## Introduction

Lung cancer (LC) remains one of the most common malignant tumors in the world, and non-small cell lung cancer (NSCLC) is the predominant type (approximately 85%) of LC, including lung adenocarcinoma (LUAC)^1^. LUAC accounts for approximately 50% of all types of LC, and its number has been increasing year by year, especially among women and young people^2^. At present, anatomical surgical resection is the most safe and effective method for patients with early-stage LUAC, including lobectomy and sublobectomy^3^. Due to the various clinical manifestations of advanced-stage LUAC, systemic and local therapies (chemotherapy, immunotherapy, radiation therapy, etc.) are considered the best option for symptom control basing on the principle of multimodal therapy^4,5^. Although LUAC’s treatment model has been greatly developed, the 5-year overall survival of patients with LUAC remains unsatisfactory (<20%)^6^. Therefore, in order to improve the early diagnosis and treatment of LUAC, it is urgent to find new biomarkers and therapeutic targets.

Unlike traditional linear RNA, circular RNA (circRNA) has a closed loop structure, which is not affected by RNA exonuclease and is not easily degraded^7^. Increasing evidence shows that circRNAs are frequently deregulated in various cancers and act as tumor suppressors or oncogenes^8^. To date, an increasing number of circRNAs have been found to function as competing endogenous RNAs (ceRNAs) in various cancers, including LUAC^9^. The ceRNA hypothesis was formally proposed in 2011 and describes a large-scale regulatory mechanism that regulates gene expression through the transcriptome^10^. MicroRNAs (miRNAs) not only affect the transcription and stability of RNA at the posttranscriptional level by binding to target genes but also affect miRNAs, which can be called “circRNA→miRNA→RNA” interactions^11^. Studies have shown that circRNA_104348 can promote hepatocellular carcinoma progression by modulating the miR-187-3p/RTKN2 (Rhotekin 2) axis^12^. Circ_0000020 can elevate the expression of PIK3CA (phosphatidylinositol 3-kinase C) and facilitate the malignant phenotypes of glioma cells by targeting miR-142-5p^13^. An increasing number of circRNAs have been found to be unbalanced in LUAC and can be used as therapeutic targets in the future. In LUAC cells, for example, hsa_circ_0027446 could promote metastasis and epithelial–mesenchymal transition (EMT), while low expression of circ_0018414 could influence proliferation, stemness, and apoptosis^14,15^. hsa_circ_0008234 is transcribed from chr3:71090478-71102924, where the FOXP1 (forkhead box P1) gene is located, approximately 587 bp in length. In gallbladder cancer, circ_0008234 contributes to tumor progression and the Warburg effect by regulating PKLR (pyruvate kinase L/R)^16^. However, there are few studies on the regulatory mechanism of circ_0008234 in LUAC.

Our research first showed that circ_0008234 was markedly decreased in LUAC tissues and cell lines. Furthermore, circ_0008234 overexpression (OE) suppressed proliferation and facilitated apoptosis in vitro and in vivo. Furthermore, circ_0008234 could sponge miR-574-5p to regulate RND3 (Rho family GTPase 3) to affect the progression of LUAC.

## Results

### The circ_0008234 expression level was decreased in LUAC tissues and cells

**Fig. 1:**
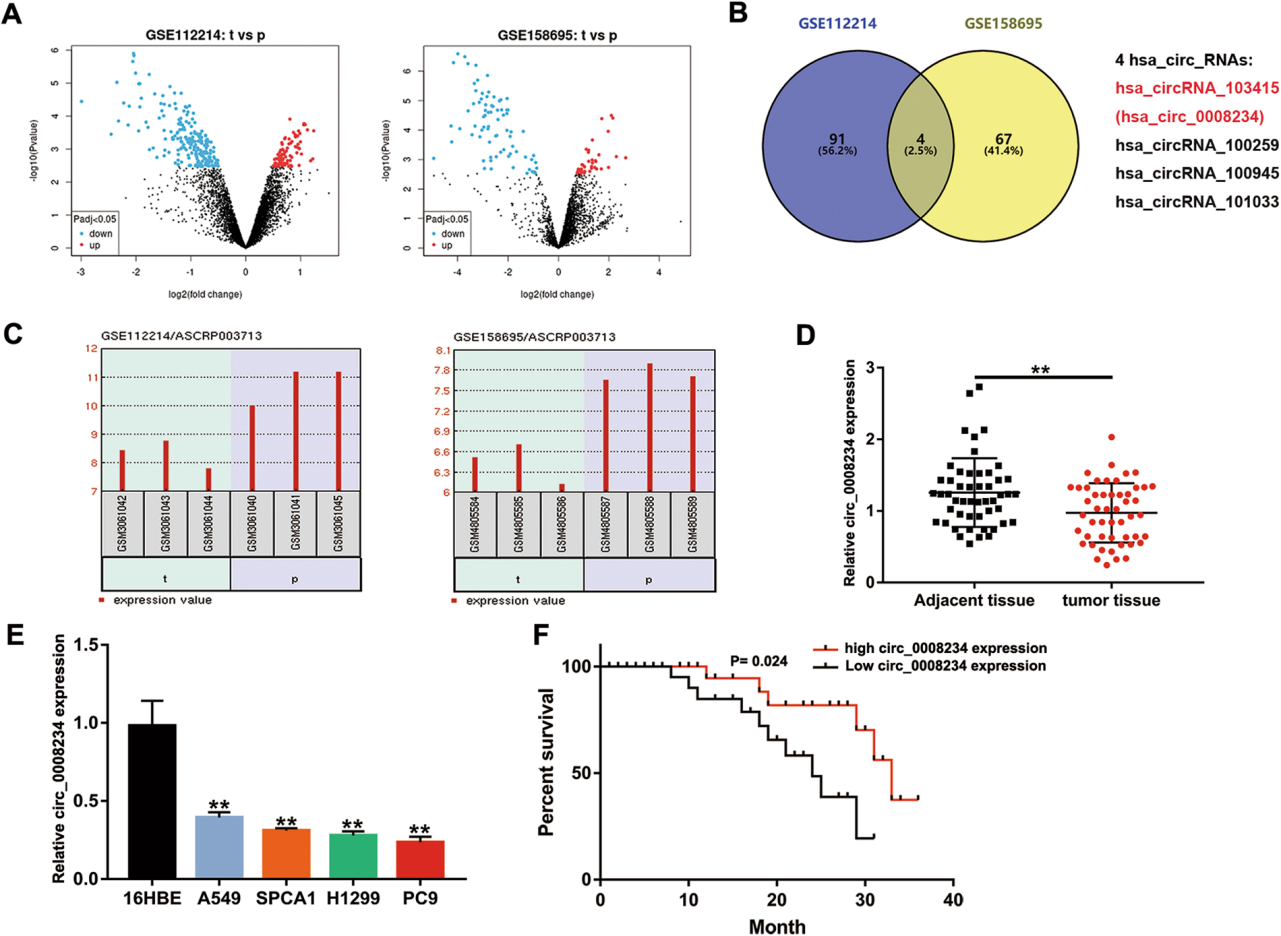
Biological characteristics of hsa_circ_0008234. **A** Original data were analyzed with GEO2R. **B** circ_0008234 was chosen from four dysregulated circRNAs. **C** circ_0008234 expression in GSE112214 and GSE158695. t tumor tissues, p pulmonary tissues; each sample record is assigned a unique and stable GEO accession number (GSMxxx). **D** Relative circ_0008234 expression in tissues from 50 LUAC patients. **E** Relative circ_0008234 expression in LUAC cells and 16HBE cells. **F** LUAC patients with high expression of circ_0008234 have a better prognosis. Data are expressed as mean ± SD, **P < 0.01.

Raw microarray data of NSCLC patients were downloaded from Gene Expression Omnibus (GEO), including GSE112214 (*n* = 6) and GSE158695 (*n* = 6). To obtain differentially expressed circRNAs, data were analyzed with GEO2R (Fig. 1A). Since there was no intersection of abnormally upregulated circRNAs, we chose circ_0008234 as the research object from the intersection of abnormally downregulated circRNAs (Fig. 1B). Subsequently, we extracted the original data of circ_0008234 from GSE112214 and GSE158695 and further verified that circ_0008234 expression in tumor tissues of NSCLC patients was observably lower than that in normal tissues (Fig. 1C). To further investigate whether this phenomenon exists in LUAC, 50 patients who met the enrollment criteria were randomly selected, and it was found that the expression of circ_0008234 in the tumor tissues was lower than that in adjacent tissues (Fig. 1D). Furthermore, circ_0008234 was significantly abnormally downregulated in the LUAC cell lines (H1299, SPCA1, A549, and PC9) compared with the 16HBE cell line (Fig. 1E). Finally, the correlations between circ_0008234 expression levels and clinical and pathological features were considered. It was found that circ_0008234 expression was remarkably correlated with the tumor size of LUAC patients (Table 1). The prognosis of LUAC patients with high expression of circ_0008234 was better than that of patients with low expression (Fig. 1F). In conclusion, circ_0008234 was downregulated in LUAC, and low expression of circ_0008234 predicted poor prognosis in LUAC patients.

**Table. 1:** Expression of circ_0008234 according to patients’ clinical features.

Factors	circ_0008234 expression		*P* value
	High	Low	
Gender			
Male	12	13	0.568
Female	10	15	
Age (years)			
≥50	8	17	0.087
<50	14	11	
Distant metastasis			
Yes	5	7	0.851
No	17	21	
Tumor size (cm)			
>3	7	18	0.023*
≤3	15	10	
Lymph node metastasis			
Yes	8	8	0.558
No	14	20	

*P < 0.05

### The biological characteristics of circ_0008234

**Fig. 2:**
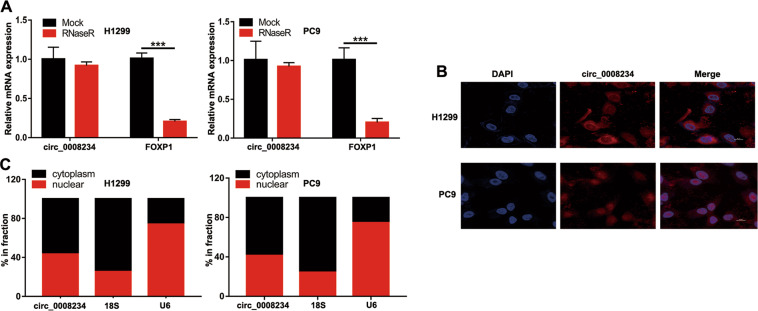
Subcellular localization of has_circ_0008234. **A** After RNase R treatment, circ_0008234 expression levels and FOXP1 mRNA expression levels in H1299 and PC9 cells were measured. **B** Immunofluorescence localization of circ_0008234. **C** Nuclear and cytoplasmic mRNA fraction experiments display the location of circ_0008234 in H1299 and PC9 cells. Data are expressed as mean ± SD, ***P < 0.001.

Based on the circ_0008234 expression of LUAC cell lines, H1299 and PC9 cells were selected for further study. In the RNase R treatment assay, the mRNA expression of FOXP1 was significantly decreased, while the circ_0008234 expression was not significantly changed (Fig. 2A). This study showed that circ_0008234 is robust and has stable expression in the cells. Then determining the subcellular location of circ_0008234 became a top priority. Next, we studied the localization of circ_0008234 in LUAC cells. Figure 2B, C showed that circ_0008234 was present in the cytoplasm and nucleus of H1299 and PC9 cells. The implication of these results was that circ_0008234 could stably exist in the cytoplasm and nucleus of LUAC cells, providing a basis for its biological functions.

### circ_0008234 OE influenced cell proliferation and apoptosis

**Fig. 3:**
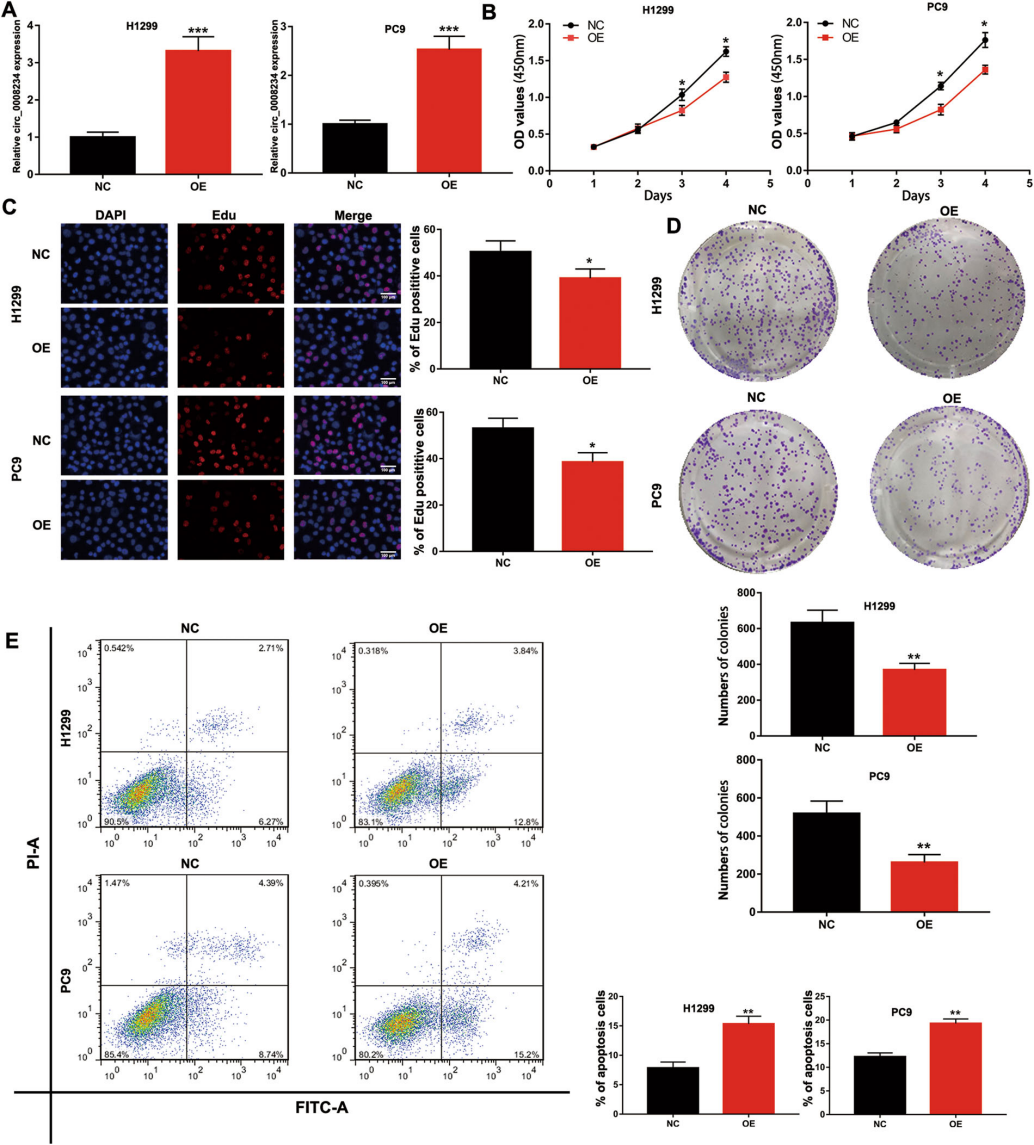
Upregulation of circ_0008234 suppressed cell proliferation and promoted apoptosis. **A** The expression levels of circ_0008234 in H1299 and PC9 cells transfected with OE-circ_0008234. **B** The proliferation capacities of H1299 and PC9 cells transfected with OE-circ_0008234 were detected by CCK-8 assays. **C** Upregulation of circ_0008234 inhibited the growth of H1299 and PC9 cells, as determined by EdU assay. **D** circ_0008234 overexpression inhibited colony formation of LUAC cells. **E** circ_0008234 promoted cell apoptosis, as shown by flow cytometry. Data are expressed as mean ± SD, *P < 0.05, **P < 0.01, ***P < 0.001.

To discover the role of circ_0008234, circ_0008234 OE plasmids and negative control (NC) plasmids were used to exogenously manipulate the expression of circ_0008234 in H1299 and PC9 cells. As shown in Fig. 3A, quantitative reverse transcriptase–PCR (qRT-PCR) results indicated that OE could specifically increase the expression of circ_0008234. On the one hand, the Cell Counting Kit-8 (CCK-8) assay reflected that the growth ability of LUAC cells was obviously decreased based on high circ_0008234 expression (Fig. 3B). On the other hand, as 5-ethynyl-2′-deoxyuridine (EdU) assays suggested, the proliferation rate of H1299 and PC9 cells was decreased by enhancing circ_0008234 expression (Fig. 3C). As shown in Fig. 3D, upregulation of circ_0008234 also reduced the colony formation ability of LUAC cells. Furthermore, circ_0008234 OE promoted apoptosis in H1299 and PC9 cells (Fig. 3E). Finally, an orthotopic xenograft mouse model was applied to determine the effect of circ_0008234 on tumorigenesis in vivo. The cells of the NC and OE groups were subcutaneously inoculated into the armpits of female nude mice. Nude mice were sacrificed 24 days later and photographed (Fig. S1A). After measuring the growth and volume of the tumor, we found that, compared with the NC group, both tumor growth and volume in the OE group were slower and smaller (Fig. S1B, C). The expression of circ_0008234 in the OE group was notably increased by using qRT-PCR (Fig. S1D). Further research also confirmed that, compared with the NC group, the positive rate of Ki67 in the OE group decreased, while the positive rate of cleavage caspase 3 increased (Fig. S1E). These experiments demonstrated that high expression of circ_0008234 inhibited proliferation and promoted apoptosis of LUAC cells.

### circ_0008234 could sponge miR-574-5p

**Fig. 4:**
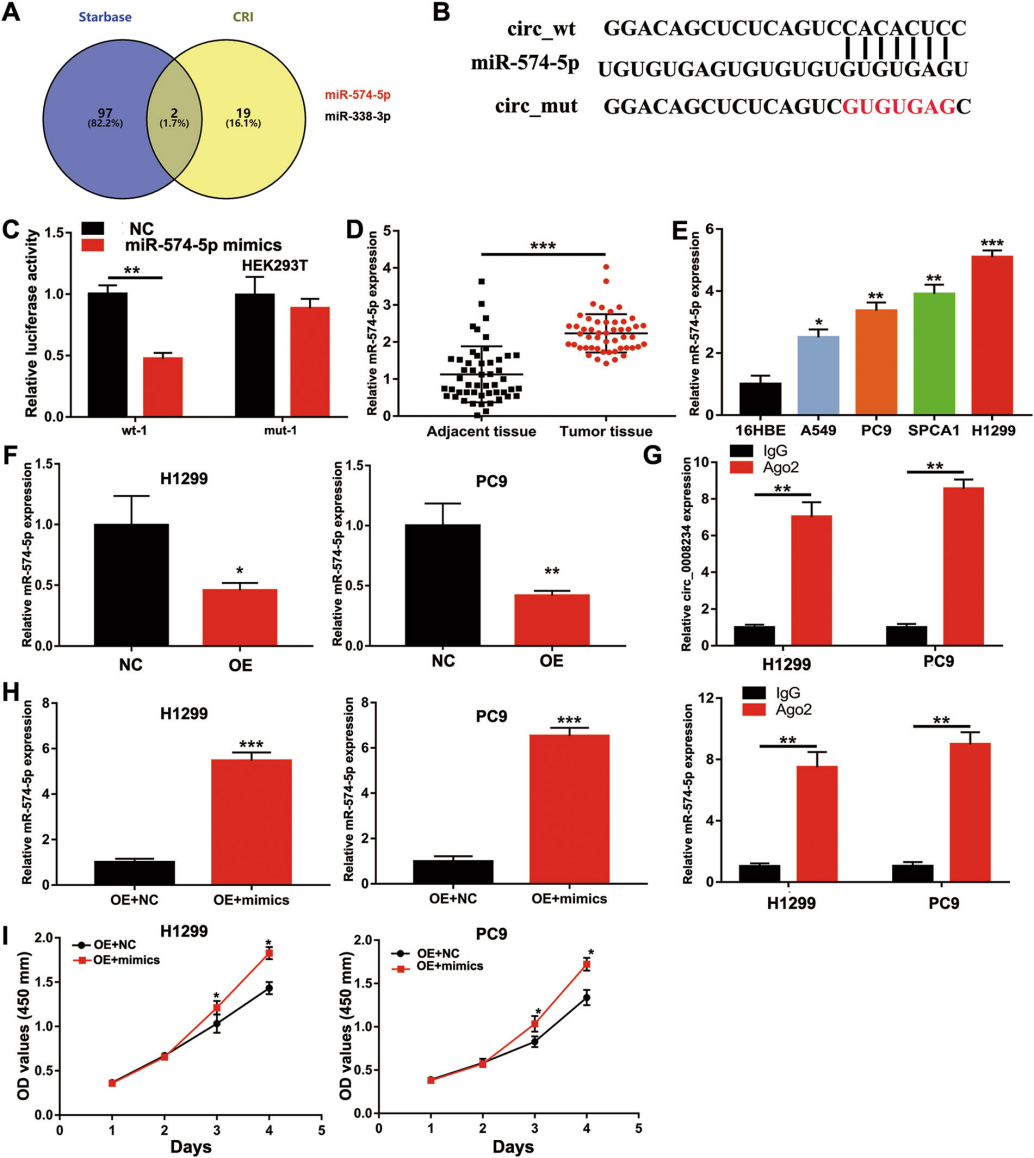
circ_0008234 may act as the miR-574-5p sponge. **A** Potential targets were screened through starBase 2.0 and CRI. **B** Schematic diagram of the binding sites between miR-574-5p and circ0008234. **C** The relative luciferase activity detected by the dual-luciferase reporter assay. **D** Relative miR-574-5p expression in tissues from 50 LUAC patients. **E** Relative miR-574-5p expression in LUAC cells and 16HBE cells. **F** miR-574-5p expression level in H1299 and PC9 cells after transfection with OE-circ_0008234 or NC. **G** RIP and qRT-PCR assays measured the expression differences in circ_0008234 and miR-574-5p between Ago2 immunoprecipitation and IgG immunoprecipitation. **H** miR-574-5p expression level in transfected H1299 and PC9 cells. **I** Overexpression of miR-574-5p accelerated cell growth, as determined by CCK-8 assay. Data are expressed as mean ± SD, *P < 0.05, **P < 0.01, ***P < 0.001.

To further show the potential mechanism of circ_0008234, miR-574-5p was chosen from starBase 2.0 and Circular RNA Interactome (CRI; Fig. 4A). The predicted binding sites between circ_0008234 and miR-574-5p are shown in Fig. 4B. To verify whether circ0008234 could directly bind to miR-574-5p, luciferase reporter gene plasmids were constructed including the circ0008234 wild-type sequence (wt-1) or mutant-type sequence (mut-1). The miR-574-5p mimics remarkably reduced the luciferase intensity of wt-1 compared to mut-1 (Fig. 4C). Furthermore, as indicated in Fig. 4D, E, miR-574-5p expression was also notably higher in LUAC tissues and cell lines. When the expression of circ_0008234 increased, miR-574-5p expression was remarkably downregulated (Fig. 4F). Then RNA immunoprecipitation (RIP) analysis showed that, compared with immunoglobulin G immunoprecipitation, circ_0008234 and miR-574-5p were remarkably enriched in Ago2 immunoprecipitation (Fig. 4G). In vitro functional assays showed that the growth rate of H1299 and PC9 cells after miR-574-5p mimic transfection was remarkably accelerated (Fig. 4H).

### RND3 may be a potential target of miR-574-5p

**Fig. 5:**
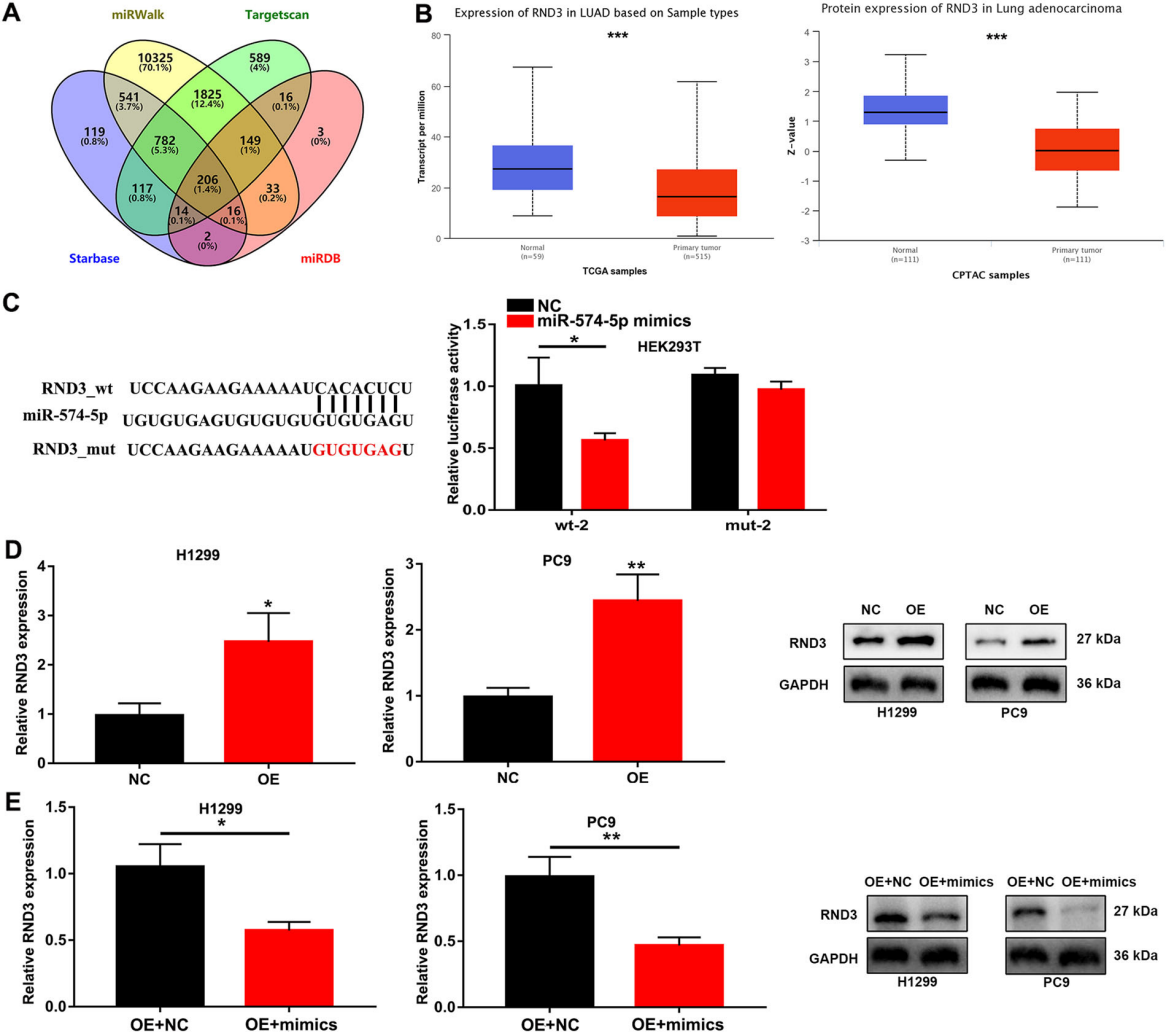
RND3 may be a potential target of miR-574-5p. **A** Potential targets were screened through starBase 2.0, miRWalk, TargetScan, and miRDB. **B** The expression of RND3 was verified through TCGA and CPTAC databases. **C** The relative luciferase activity detected by the dual-luciferase reporter assay. **D** RND3 mRNA and protein expression in H1299 and PC9 cells transfected with OE-circ_0008234. **E** RND3 mRNA and protein expression in transfected H1299 and PC9 cells. Data are expressed as mean ± SD, *P < 0.05, **P < 0.01, ***P < 0.001.

To determine the potential target of miR-574-5p, starBase 2.0, TargetScan, miRWalk, and miRDB were used, and 206 genes were found (Fig. 5A). After verification by The Cancer Genome Atlas (TCGA) and Clinical Proteomic Tumor Analysis Consortium (CPTAC) databases, RND3 was finally chosen (Fig. 5B). Another dual-luciferase reporter gene assay verified the possibility of complementary base pairing between miR-574-5p mimics and RND3 mRNA (Fig. 5C). At the same time, after OE of circ_0008234 in H1299 and PC9 cells, the mRNA and protein expression of RND3 was significantly upregulated (Fig. 5D). As shown in Fig. 5E, the mRNA and protein expression of RND3 in LUAC cells also decreased when miR-574-5p was overexpressed. These results suggested that circ_0008234 may regulate RND3 by sponging miR-574-5p.

## Discussion

Despite various advances and achievements in the diagnosis and treatment of LUAC, it remains one of the main causes of death globally^17^. The number of NSCLC cases has increased year by year, and the incidence of LUAC is the highest^18^. A growing number of studies have suggested that dysregulated circRNAs act as regulators in diverse diseases, including LUAC^19–21^.

In this manuscript, we identified a dysregulated circRNA (circ_0008234) in LUAC tissues through the GEO database. Low expression of circ_0008234 was first observed in LUAC tissues and cell lines. The expression level of circ_0008234 was closely correlated with tumor size, and circ_0008234 expression may be a predictor of prognosis. Functionally, circRNAs may play regulatory roles in LUAC. As recently reported, circRNA disorders can cause malignant behavior in human cancer cells^22^. Then in vitro and in vivo assays were designed and conducted to detect the proliferation and apoptosis rate of circ_0008234 in LUAC cell processes. After cell transfection, we determined that circ_0008234 OE would impede cell proliferation and accelerate apoptosis in LUAC.

Further research found that circ_0008234 could enhance the RND3 expression by modulating miR-574-5p. miR-574-5p, as a member of the microRNA family, is closely tied to the occurrence and development of cancers. For example, miR-574-5p regulated cell invasion by regulating FOXN3 (forkhead box N3) in nasopharyngeal carcinoma and attenuated proliferation, migration, and the EMT pathway by modulating BCL11A (BAF chromatin remodeling complex subunit BCL11A) and SOX2 (SRY-box transcription factor 2) in triple-negative breast cancer^23,24^. Furthermore, miR-574-5p was noticeably increased in the serum and tissue samples of early-stage NSCLC patients and promoted metastasis by modulating PTPRU (protein tyrosine phosphatase receptor type U)^25,26^. In addition, the expression of miR-574-5p is regulated by long non-coding RNA (lncRNA) in cancers. For example, lncRNA PTCSC3 inhibited cervical carcinoma cell proliferation and invasion by sponging miR-574-5p, and LINC00052 inhibited metastasis by regulating miRNA-574-5p in colorectal cancer^27,28^. However, the sponge adsorption effect of circRNA on miR-574-5p has not been studied. In this study, miR-574-5p was confirmed to be upregulated in LUAC tissues and cell lines, and circ_0008234 played a role as a tumor-suppressor gene by downregulating miR-574-5p. Through dual-luciferase reporter gene assay and RIP assay, it was verified that circ_0008234 and miR-574-5p underwent direct complementary base pairing. Hence, we concluded that circ_0008234 led to a significant downregulation of miR-574-5p.

Next, we also found a predicted target gene downstream of miR-574-5p: RND3. RND3 is a member of the RhoGTPase family and belongs to the RND subfamily, also known as RhoE^29^. RND3 participates in functions frequently regulated by Rho-GTPase, such as remodeling of the actin cytoskeleton, and numerous essential cellular processes, such as cell proliferation, differentiation, survival, motility, and adhesion^30,31^. These mechanisms may contribute to cancer development when dysregulated. Early research has shown that RND3 is overexpressed in NSCLC patients and is interconnected with poor prognosis, indicating that RND3 can be chosen as a prognostic marker for NSCLC patients^32,33^. However, recently generated functional data indicated that RND3 was downregulated in three NSCLC cell lines (one of which was a LUAC cell line), and the enhanced expression of RND3 inhibited cell proliferation^34^. The evaluation of RND3’s impact on LUAC requires more in-depth research. In this manuscript, we verified the low expression of RND3 in LUAC tissues through the TCGA and CPTAC databases. Another dual-luciferase reporter assay indicated that there was a binding phenomenon between miR-574-5p and RND3 mRNA. The expression of RND3 mRNA was significantly upregulated when circ_0008234 was overexpressed. However, there was a negative relationship between the expression of RND3 and miR-574-5p. In summary, the above-mentioned results indicated that circ_0008234 could decelerate LUAC progression by serving as a miRNA sponge to regulate miR-574-5p RND3 expression.

Our manuscript highlights the potential of circ_0008234, miR-574-5p, and RND3 as novel therapeutic targets for treating LUAC patients and may help to further explore the specific mechanism of the ceRNA network in cancer. In future studies, we will combine these results with the application of traditional chemotherapy drugs or molecular-targeted antitumor drugs surgical patients or advanced patients to identify surgical opportunities to improve the prognosis and survival rate.

## Conclusion

Our data indicate that circ_0008234 has a key part in the occurrence and development of LUAC, mainly through the “sponge effect” to modulate miR-574-5p expression, thereby promoting the mRNA expression of RND3. This research will provide new directions for improving and expanding molecular targets for the diagnosis and treatment of LUAC.

## Materials and methods

### Bioinformatics analysis

Microarray data were downloaded from the GEO database^35^, including GSE112214 and GSE158695^36^. Then the original microarray data were analyzed with GEO2R. All information on hsa_circ_0008234 comes from circBase (http://www.circbase.org/)^37^. The binding site information of miRNAs and circ_0008234 was derived from starBase 2.0 (http://www.starbase.sysu.edu.cn/starbase2/) and CRI (http://circinteractome.nia.nih.gov/)^38,39^. The binding site information of microRNA-574-5p and mRNAs was derived from starBase 2.0 (http://www.starbase.sysu.edu.cn/starbase2/), TargetScan (http://www.targetscan.org/), miRWalk (http://mirwalk.umm.uni-heidelberg.de/), and miRDB (http://mirdb.org/policy.html)^38,40–42^. TCGA and CTPAC were used to identify the expression of RND3 between tumor tissues and adjacent tissues^43,44^.

### LUAC tissues and cell lines

Fifty tissue samples of LUAC patients have been obtained from the First Affiliated Hospital of Soochow University since June 2019. The clinical characteristics of the patients were recorded based on the American Joint Committee on Cancer tumor/node/metastasis staging system (eighth edition). None of these patients received radiation or chemotherapy before surgical resection. LUAC cell lines (A549, SPCA1, H1299, and PC9), the 16HBE14o Human Bronchial Epithelial Cell Line (16HBE), and HEK293T cells were sourced from the Shanghai Academy of Sciences (Shanghai, China). All cells were moistened in RPMI 1640 medium (Gibco, NY, USA) supplemented with 10% fetal bovine serum (Gibco) and 100 U/mL penicillin and streptomycin (Gibco). Cells were cultured in an incubator under a 5% CO_2_ atmosphere at 37 °C.

### Cell proliferation and apoptosis assay

CCK-8, EdU, colony formation, and apoptosis assays were performed as described previously^45^.

### Statistical analysis

Statistical analyses were performed by using the SPSS 25.0 software (IBM Corp., NY, USA). All means and standard deviations were calculated based on three independent experiments. Correlations between circ_008234 expression and the clinicopathological characteristics of the LUAC patients were examined using the *χ*^2^ test. Comparisons between two groups were analyzed using a two-sided Student’s *t* test. *P* < 0.05 was considered statistically significant.

More details can be found in S2.

## Supplementary information

Figure. S1

Supplementary figure legend

## Data Availability

Data supporting the present findings are available from the corresponding author upon reasonable request.
