# Supplementary figures and images for: hsa_circ_0008234 inhibits the progression of lung adenocarcinoma by sponging miR-574-5p
Source: Cell Death Discov. 2021 May 28;7:123. doi: 10.1038/s41420-021-00512-1 (PMC8163831; doi:10.1038/s41420-021-00512-1)

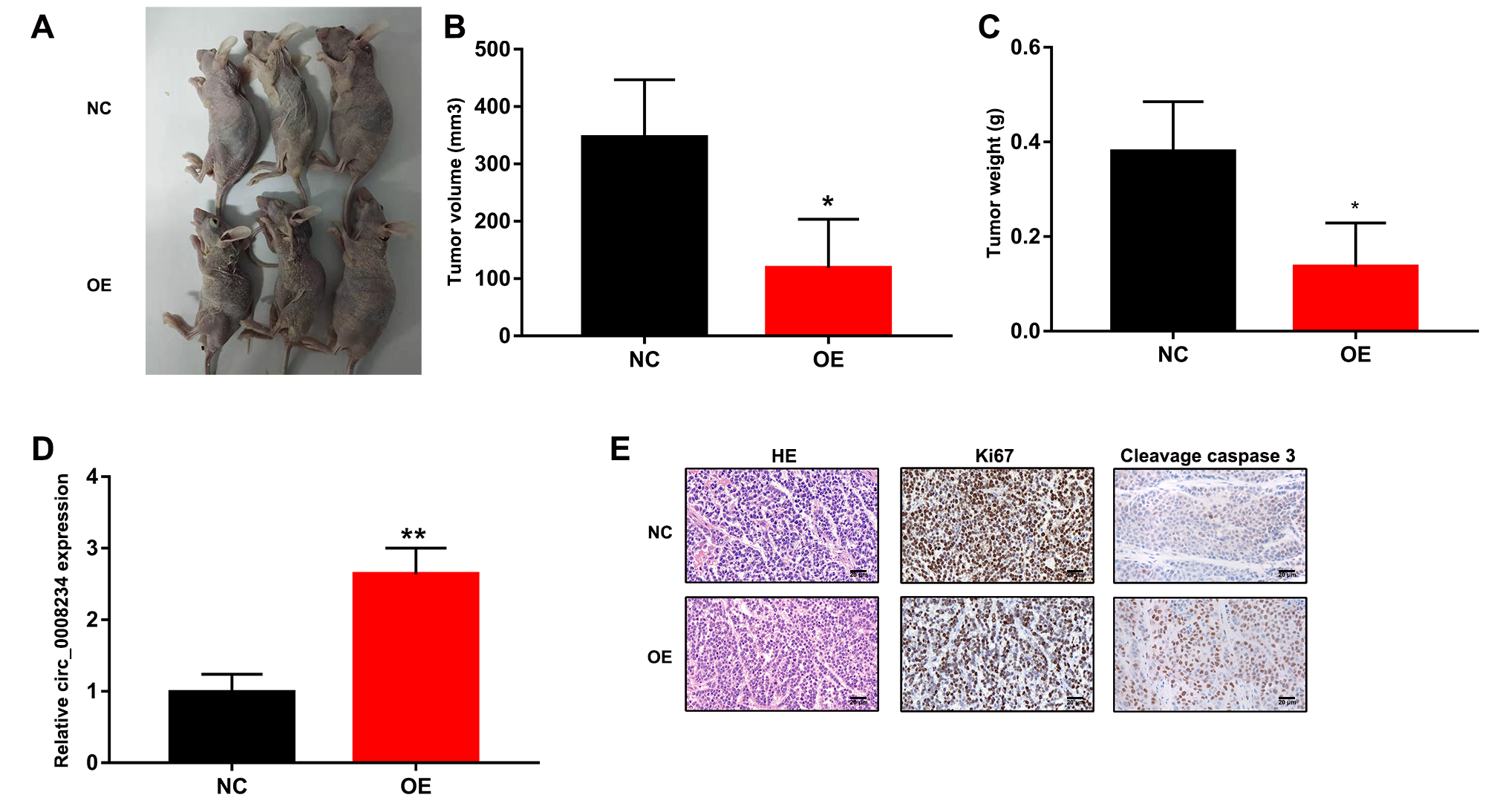

Supplement: Supplementary file 1 — Figure. S1 [file 41420_2021_512_MOESM1_ESM.tif]
