# Supplementary material for: hsa_circ_0008234 inhibits the progression of lung adenocarcinoma by sponging miR-574-5p
Source: Cell Death Discov. 2021 May 28;7:123. doi: 10.1038/s41420-021-00512-1 (PMC8163831; doi:10.1038/s41420-021-00512-1)
Supplement: Supplementary file 2 — Supplementary figure legend [file 41420_2021_512_MOESM2_ESM.docx]

**Fig. S1. The effect of circ_0008234 *in vivo*. (A)** circ_0008234 overexpression reduced tumorigenicity in nude mice. **(B)** The tumor volumes were smaller in the OE group than in the NC group. **(C)** The tumor weights were lighter in the OE group than in the NC group. **(D)** The circ_0008234 expression level was upregulated in the OE group compared with the NC group. **(E)** The results of HE and IHC. Data are expressed as the mean ± SD, **P < 0.05, **P < 0.01.*
